# Spontaneous Fluctuations in Alpha Peak Frequency along the Posterior-to-Anterior Cortical Plane

**DOI:** 10.1523/ENEURO.0118-25.2025

**Published:** 2026-01-02

**Authors:** Vaishali Balaji, Alfons Schnitzler, Joachim Lange

**Affiliations:** Institute of Clinical Neuroscience and Medical Psychology, Medical Faculty and University Hospital Düsseldorf, Heinrich Heine University Düsseldorf, Düsseldorf 40225, Germany

**Keywords:** alpha oscillations, alpha peak frequency, cortical gradients, magnetoencephalography

## Abstract

Alpha peak frequency (APF) is defined as a prominent spectral peak within the 8–12 Hz frequency range. Typically, an individual's alpha frequency is regarded as a stable neurophysiological marker. A wealth of recent evidence, however, indicates that APF shifts within short timescales in relation to task demands and even spontaneously so. Further, brain stimulation studies often report shifts in APF both within and between experimental sessions, directly contradicting the idea of a stable APF. To characterize the nonstationarities in spectral parameters, we estimated APFs from 1 s epochs of resting-state magnetoencephalography (MEG) recordings from healthy adults of either sex. To enhance signal-to-noise ratio, without compromising on temporal resolution, we averaged power spectra within parcelled regions. Our findings indicate that variation in APFs exacerbates along the posterior-to-anterior cortical plane, i.e., from the occipital to the frontal cortices. Further, by comparisons with amplitude-matched simulated signals, we demonstrated that the observed gradient is not attributable to measurement noise. Across the cortex, APFs showed poor temporal reliability, raising the question of whether APFs are more like a transient state than a trait. In general, our study elucidates the dynamic characteristics of alpha oscillations and reveals systematic regional differences which are, in part, shaped by underlying signal-to-noise ratio inherent to MEG recordings.

## Significance Statement

Oscillatory signals, as recorded with electro-/magnetoencephalography, exhibit a prominent peak in the alpha frequency range (i.e., APF). It is widely accepted that the amplitude and phase of oscillatory signals vary with behavior. However, the stability of APF within and across experimental sessions is seldom examined. In this study, we characterized the changes in APF to show that the degree of fluctuations in APF systematically increased from the occipital cortex to the frontal cortex, forming a gradient across the cortical surface. We also established that the observed variability was not driven by underlying noise. Our results raise the possibility that the dynamics of APF could be a salient feature of a cortical region, driven by its underlying structure and function.

## Introduction

Alpha oscillations, within the 8–12 Hz frequency range, are a prominent feature of electro- and magnetoencephalography (E/MEG) recordings (Berger, 1929). Parameters of the alpha oscillations, such as power and phase, have been studied extensively in the context of neural inhibition and excitation (Klimesch et al., 2007; Jensen and Mazaheri, 2010; Jensen et al., 2012; Lange et al., 2013; VanRullen, 2016). Typically, these studies examined power and phase in fixed or predefined frequencies or frequency bands. However, the practice of employing strict, a priori defined, frequency bands might overlook potentially meaningful variation within the alpha band (Tallon-Baudry, 1999; Jones et al., 2009; Sherman et al., 2016; Donoghue et al., 2022). Alpha peak frequency (APF), defined as the frequency having the maximum power, is a distinct measure of oscillatory activity. As APF is estimated per individual from the empirical data, it allows for flexible comparisons of intra- and interindividual differences in behavior and cognition (Baumgarten et al., 2023).

Especially within the context of perception, APF has been widely implicated in the speed of processing information. APF is said to determine the temporal resolution of the visual system whereby fluctuations in APF dictate whether one perceives discrete visual stimuli as simultaneous or separate (Samaha and Postle, 2015; Drewes et al., 2022; Sharp et al., 2022). Similarly, top-down modulation of APF was demonstrated in the context of a working memory paradigm with an increase in APF in conjunction with increased memory load (Haegens et al., 2014; Babu Henry Samuel et al., 2018). Even in the absence of experimental manipulation, Benwell et al. (2019) found a difference in APF between two halves of an experimental session, whereas Cohen (2014) reported shifts in APFs within resting-state networks. Further, Mahjoory et al. (2020) showed a systematic decrease of APF across the cortex in the posterior to anterior direction, suggesting that APF varies spatially.

Contradictorily, it is assumed that APF is a stable “trait” marker (Grandy et al., 2013; Haegens et al., 2014). This view is mainly driven by the longitudinal changes in APF across the lifespan in congruence with brain volume (Lindsley, 1939) and relative stability over test–retest intervals (Kondacs and Szabó, 1999) despite cognitive interventions (Grandy et al., 2013). APF is considered a neurophysiological fingerprint, especially due to its presumed heritability (Smit et al., 2006) and marked interindividual differences (Klimesch, 1999; Posthuma et al., 2001). However, APF may resemble a state factor depending on the timescale of the investigation. Conventional analyses, which estimate APF from 2 to 5 min of resting-state M/EEG, capture the mean peak frequency over this time period; however, the APF may spontaneously fluctuate around the mean frequency in response to changes in physical stimuli or cognitive state. In fact, numerous noninvasive brain stimulation studies report the notoriety of matching stimulation frequency to APF (Vossen et al., 2015; Stecher and Herrmann, 2018), highlighting the practical pitfalls of assuming that APF remains stationary over time.

Findings from computational studies collectively suggest that frequency transitions within the alpha band are likely a natural consequence of variation in synaptic input and concurrent firing rates of neural populations (Lefebvre et al., 2015; Hutt et al., 2016). When firing rates increase relative to baseline, cortical networks recruit nonlinear feedback loops, in turn changing the system's response function and the features of its emergent dynamics (Mierau et al., 2017). Therefore, APF is a proxy for the activation state of a population of neurons. As such, the fluctuations of frequency are a dynamic signature of low-frequency neural oscillations, but they are rarely accounted for in the analysis of experimental data.

Building on previous work by Smith et al. (2024), we aimed to analyze the stability of APF at short timescales. To this end, we estimated individual APFs from source-reconstructed power spectra derived from brief time series (1 s epochs) of resting-state magnetoencephalography (MEG). We decomposed the power spectrum into periodic and aperiodic components, ensuring that estimates of APF are unaffected by aperiodic activity. Since frequency of the intrinsic oscillator may vary spontaneously in conjunction with synaptic input, we hypothesized that APFs would show considerable variability within individuals. Further, brain areas vary in terms of spectral profiles (Keitel and Gross, 2016; Mellem et al., 2017; Mahjoory et al., 2020), developmental trajectories (Charvet and Finlay, 2014), and structural connections (Huntenburg et al., 2017); hence, we hypothesized that the variability of APFs would also be spatially heterogeneous.

## Materials and Methods

### Experimental design

In this study, we analyzed a previously acquired dataset (manuscript has been submitted for publication), containing ∼4 min of resting-state MEG recordings from 24 healthy participants (11 female, age 25.33 ± 2.81 [mean ± SD] years). The participants were seated upright in a dimly lit magnetically shielded room while we recorded neural activity using a 306-channel MEG system, with a sampling frequency of 1,000 Hz (MEGIN Oy).

The MEG recordings are from a study where transcranial alternating current stimulation (tACS) was applied to the participants’ somatosensory cortex. Each recording started with a baseline period of 2 min, followed by periods of either 10 or 30 s of tACS, or no stimulation (control). The data used in the present study were collected during the control session. That is, tACS electrodes were placed at the participants’ scalp, but no electrical stimulation was administered. The participants were unaware of the stimulation condition (10 s, 30 s, or control). Hence, we classify this dataset as resting-state MEG.

In addition, we instructed participants to focus on a white fixation cross through the duration of the experiment. The cross was projected (PT-DW700E, Panasonic) on translucent screen placed ∼57 cm in front of the participants. The fixation cross rotated by 45° for 500 ms at random time points. Participants had to press a button within 2 s of the rotation. This task served merely as a vigilance task paradigm and was included to ensure sustained attention (Zaehle et al., 2010).

We acquired anatomical T1-weighted MRIs with a 3T Siemens scanner (Siemens Magnetom Tim Trio 3T, Siemens). We used the Polhemus Fastrak system (Polhemus) to digitize HPI coils, fiducial markers, and ∼50–100 additional points. We recorded electrooculogram (EOG) to monitor blinks and eye movements.

### MEG data preprocessing

Entire preprocessing and analysis of the datasets were performed using the FieldTrip toolbox (version 20240614) and custom-made scripts on MATLAB (R2023b; The MathWorks).

We segmented the continuous MEG recording into trials, consisting of a single baseline trial (110 s) and 20 resting-state trials (of ∼5.5 s). We restricted further preprocessing to 204 gradiometer channels. We applied a bandpass filter of 4–150 Hz and removed the linear trends and mean of every trial. We applied FieldTrip's semiautomatic artifact detection approach to remove segments of trials containing artifacts (e.g., SQUID jumps, head and muscle movements). After visual inspection, we additionally removed noisy channels and trials containing artifacts. We used Independent Component Analysis (ICA) to remove any remaining electro-cardio and electro-ocular artifacts.

### Source reconstruction

We reconstructed the sources of the time-series data measured by MEG sensors using Linearly Constrained Minimum Variance (LCMV) beamformers (Van Veen et al., 1997).

We generated a single-shell volume conduction model by performing coregistration of the sensors to participants’ anatomical MRIs using fiducial markers, followed by segmentation of the brain surface (Nolte, 2003). Then we divided the brain volume into a regular 3D grid with a 5 mm resolution, based on the MNI template brain (Montreal Neurological Institute).

We restricted the analysis to cortical regions resulting in 8,384 grid points. We estimated spatial filters for each of the cortical grid points along the dipole direction with maximal power, with a regularization parameter of 10%. By multiplying these filters with sensor-level data, we reconstructed time-series data at the source. Subsequently, source-level time-series were parcellated into 210 cortical regions of interest based on the Brainnetome Atlas (Fan et al., 2016).

### Data analysis

We concatenated baseline and resting-state trials to create a continuous segment of the time series (∼220 s) and then segmented parcel time-series into 1 s epochs. We computed power spectra for each epoch using a multitapered (3 tapers) fast Fourier transform, based on discrete prolate spheroidal sequences (dpss), with 2 Hz spectral smoothing. We averaged power spectra over grid points within each parcel, resulting in ∼220 power spectra per parcel and participant. We estimated the aperiodic 1/f component using the “Fitting Oscillations One Over F” (FOOOF; Donoghue et al., 2020) algorithm in Python 3.7.2. Then we subtracted the 1/f component from the corresponding spectra and determined the spectral peaks in the alpha band (7–14 Hz), for each corrected spectrum of a parcel, using the “*findpeaks*” function on MATLAB. The frequency of the peak with the strongest power was regarded as the APF of a given spectrum. Epochs without a definitive spectral peak, i.e., a flat peak with two neighboring frequencies of the same power, were given the value of a “NaN.”

### Statistical analysis

First, we sought to replicate the results of Mahjoory et al. (2020). To this end, we performed a linear regression between time-averaged APF of 210 parcels and their corresponding *y*-coordinate position, along the posterior-to-anterior direction. Second, to quantify the degree of variation in APFs within participants, we calculated the coefficient of variation (CV), for a given parcel, by dividing the standard deviation by the mean APF across 220 epochs. For a given parcel, *k*:

$$CV_{\mathrm{parcel}\;k}\;=\sigma_{\mathrm{epochs}\;k}÷\mu_{\mathrm{epochs}\;k}.$$

To test the regional differences in CV along the posterior-anterior axis, we performed a linear regression between CV values of all parcels and their corresponding *y*-coordinate position.

To check whether the spatial profile of alpha power confounded the estimation of APF, we calculated signal-to-noise ratio (SNR) and CV for each of the 8,384 cortical grid points. We defined SNR as the ratio between the power of the APF and the average power of spectral peaks in all other frequencies. We averaged SNR and CV values within a spatial sliding window of 2.5 cm in increments of 0.25 cm (along the posterior to anterior direction), resulting in 61 SNR and CV values per participant. We concatenated the CV and SNR values across participants and performed Pearson’s correlation.

To contrast the variation in APF with noise, we subjected 20 empty-room datasets to the same preprocessing and data analysis steps as detailed in the foregoing sections. Additionally, we added a 10 Hz alpha and a 20 Hz beta rhythm to the empty-room time series. The simulated alpha peaks decreased in amplitude along the posterior to anterior direction, thereby mimicking the physiological data (Extended Data Fig. 3-1). The beta rhythm was added as a proxy to neuronal noise. We obtained CV values for the 210 parcels of physiological data, CV values for 204 MEG sensor channels of non-neuronal noise data, and CV values for 204 MEG sensor channels of simulated noise data. We averaged CV values along the *y*-axis gradient using a spatially sliding window as detailed above. Then we performed independent *t* tests for each of the 61 *y*-coordinate positions between signal and noise data. To circumvent the multiple comparison problem, we performed a nonparametric cluster-based permutation test. We permuted the data by shuffling the condition assignment (signal vs noise). Spatially adjacent *y*-coordinate positions, which met a priori defined threshold (*α* = 0.05), were combined to a cluster. On each permutation, we retained the cluster with the maximum sum of *t* values. Given 1,000 summed cluster *t* values, obtained from 1,000 permutations, we determined the probability of the empirical data.

To ensure that peaks identified from the physiological data reflected genuine neural activity rather than ambient noise, we estimated the distribution of spectral peak powers across the empty-room recordings (per MEG sensor), and we discarded epochs from physiological data, if the identified APF did not exceed the 99th percentile of this noise distribution (Extended Data Fig. 3-2). We then calculated CV values for all remaining APFs per parcel. To test whether the regional differences in CV persisted after excluding APFs with low amplitudes, we performed a linear regression between the recalculated CV values and their corresponding *y*-coordinate position.

Finally, we examined whether APF is a stable “trait-like” construct which is distinguishable between subjects. We used intraclass correlation (ICC) to measure the reliability of APF which akin to intrarater reliability, quantifies the variation of data measured by one rater across two or more trials. In our study, it is the extent to which a single individual consistently produces the same APFs across epochs. Specifically, we implemented a single-rater two-way mixed-effects model and absolute agreement definition, or ICC(A,1) (Shrout and Fleiss, 1979; McGraw and Wong, 1996). Generally, ICC is calculated as a ratio:

$$\frac{MS_{B}\;-\;MS_{E}}{MS_{B}+(k-1)MS_{E}+\frac{k}{n}(MS_{W}-MS_{E})},$$

where MS*_B_* is the mean square between participants, representing the variance attributable to true differences between individuals; MS*_E_* is the mean square for error, representing the residual error variance (i.e., the variance not accounted for by participant or measurement); MS*_W_* is the mean square within participants, representing the variance between measurements; *k* is the number of measurements (or epochs); and *n* is the number of participants. If the MS*_E_* is equal to or larger than the variance of interest (MS*_B_*), the reliability of APFs is evidently poor.

ICC estimates and their 95% confidence intervals were calculated using the Matlab Central file-exchange *ICC.m* function (Salarian, 2016). This ICC calculation was applied at every parcel to obtain spatially specific reliability estimates. In addition, we estimated APFs from epochs of 2 and 5 s to chart out how ICC increases with longer periods of averaging (Extended Data Figs. 4-2, 4-3). ICC estimates range from 0 to 1, with values closer to 1 indicating higher reliability.

## Results

We successfully replicated the key results of Mahjoory et al. (2020) showing that APF decreased significantly along the posterior-to-anterior axis (*R*^2^ = 0.646, *p* < 0.001; Extended Data Fig. 1-1). The left lateral occipital cortex had the highest APF of 10.57 ± 1.61 Hz [mean ± SD], whereas the left middle frontal gyrus had the lowest APF of 7.5 ± 0.74 Hz [mean ± SD].

**Figure 1.**
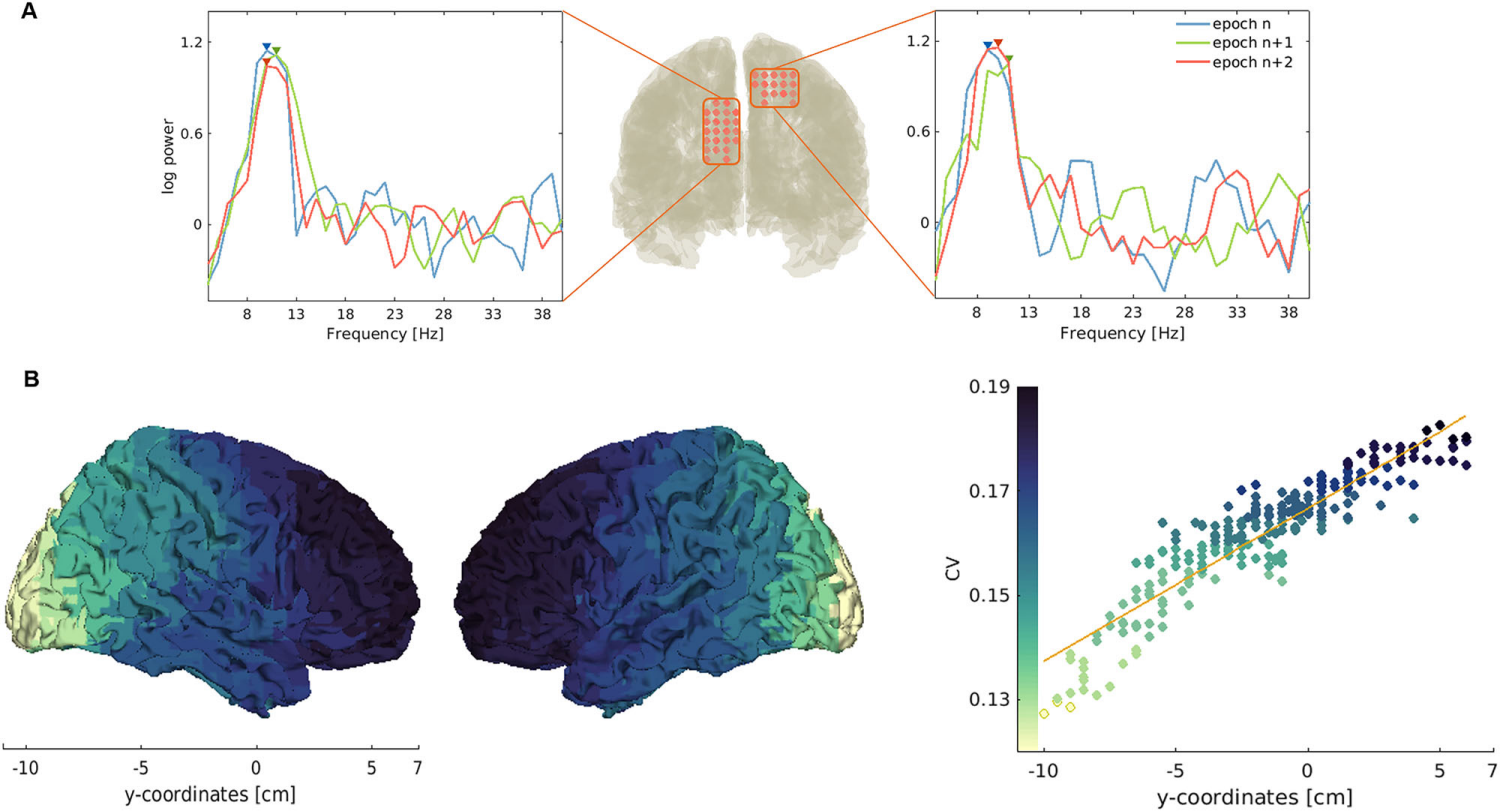
Variation in alpha peak frequency. ***A***, Exemplary demonstration of shifts in APF in three consecutive epochs. The left panel shows shifts in APF in the left lateral occipital cortex from 9 to 10 Hz, and then back to 9 Hz, all within 3 s. The right panel shows shifts in APF in the right superior parietal lobule from 9 to 11 Hz, and then to 10 Hz, again within 3 s. ***B***, In the left panel, the degree of variation in APF, measured by coefficient of variation (CV), is projected on an MNI template brain. In the right panel, CV values were averaged along a spatially sliding window in the posterior-to-anterior direction. *y*-coordinate position of the parcel significantly predicted CV (*R*^2^ = 0.841, *p* < 0.001). APF itself decreased significantly along the posterior-to-anterior axis (Extended Data Fig. 1-1).

To quantify changes in APF within participants, we calculated the CV for all cortical parcels. CV values linearly increased along the posterior-to-anterior axis (*R*^2^ = 0.841, *p* < 0.001; Fig. 1*B*, Table 1). The lateral occipital cortices showed the least amount of variation in APF, with an average CV of 0.13 ± 0.03 [mean ± SD], whereas the superior and middle frontal gyri showed the most variation, with an average CV of 0.18 ± 0.01. Among individuals, 70% showed a moderate positive correlation between *y*-coordinate position and CV (*r* > 0.6).

**Table 1.** Results of statistical tests

Figure	Comparison	Type of test	Statistic	95% CI
1*B*	CV and *y*-coordinate of parcel	Linear regression	*R*^2^ = 0.841, *p* < 0.001	[0.807,0.871]
2	CV and SNR	Pearson’s correlation	*r* = −0.87, *p* < 0.001	[−0.878, −0.861]
3*A*	CV of signal and noise	Cluster-based permutation	Cluster statistic = −241.39, *p* < 0.001	CI range: [0.002]
4	APFs of parcels	Intraclass correlation (ICC)	Right precentral: ICC = 0.121	[0.075, 0.216]
			Right superior frontal gyrus: ICC = 0.018	[0.009, 0.040]

10.1523/ENEURO.0118-25.2025.f1-1 Figure 1-1 **Distribution of time-averaged APF** In the left panel, time-averaged APF for each parcel is projected on an MNI template brain. In the right panel, APF values were averaged along a spatially sliding window in the posterior-to-anterior direction. We found that the Y-coordinate position of the parcel significantly predicted APF (R^2^ = 0.646, p < 0.001). Download Figure 1-1, TIF file.

**Figure 2.**
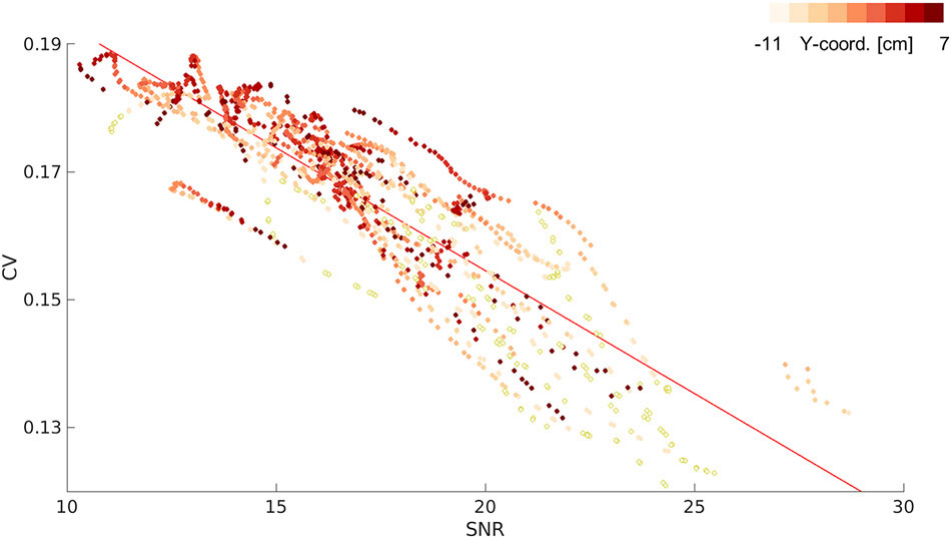
Correlation between degree of variation (CV) and signal-to-noise ratio (SNR). SNR of a parcel spectrum is the relative difference between the power of the APF and the average power of all other peak frequencies. The color bar indicates the *y*-coordinate position of each data point. Lighter colors lie caudally, whereas darker shades lie rostrally along the *y*-coordinate axis. We found a significant negative correlation between SNR and CV, such that a lower SNR was associated with a higher CV in APF (*r* = −0.87, *p* < 0.001).

We found a significant negative correlation between SNR and CV (*r* = −0.87, *p* < 0.001; Fig. 2). This suggests that low alpha power in the anterior regions may preclude the estimation of APF, resulting in seemingly high CV. Alternatively, it could also be driven by the presence of multiple alpha band oscillations of comparable magnitude occurring simultaneously in anterior regions, with APF switching between peaks as their relative prominence fluctuates.

**Figure 3.**
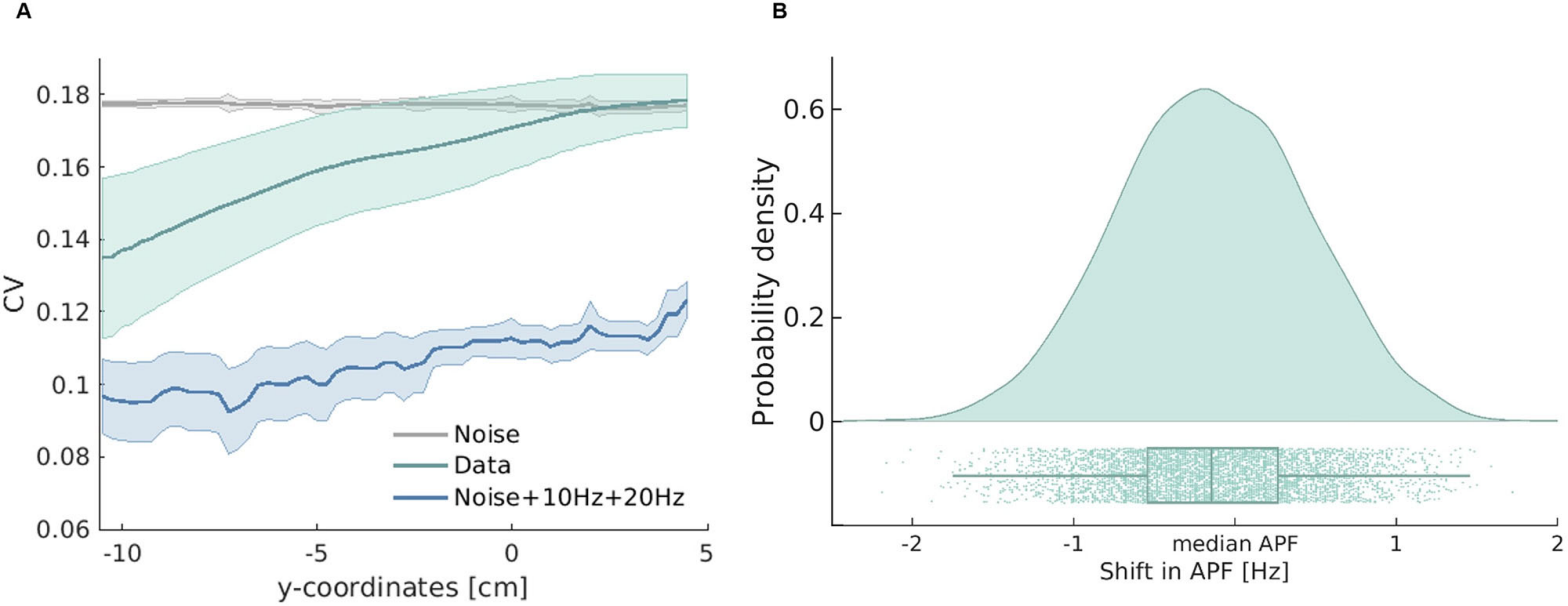
Analysis of degree of variation (CV) in relation to simulated signal and noise. ***A***, The CV of physiological signal (in teal), non-neuronal noise (in gray), and simulated noise + signal (in blue) is plotted along the *y*-coordinate plane. The shaded region indicates the standard deviation across datasets. On comparing physiological signal with empty-room noise, we found a statistically significant cluster between −10.5 and 0 cm (i.e., from the occipital cortex to the precentral gyri). On contrasting physiological signal with simulated noise (i.e., Noise +10 Hz +20 Hz), we found a statistically significant cluster across the entire cortex. Simulated alpha and beta peaks show amplitudes comparable to the physiological data (Extended Data Fig. 3-1). The linear increase in CV values along the posterior-to-anterior axis remains evident after excluding APFs that did not meet the threshold (Extended Data Fig. 3-2). ***B***, Probability density of APF, illustrating the distribution of APF shifts from the median APF of a parcel within the significant spatial cluster (between −10.5 and 0 cm).

To elucidate whether the temporal fluctuations in APF from second-to-second are simply a by-product of noise, we contrasted APFs estimated from physiological data with APFs estimated from empty-room measurements. CV of physiological signal was significantly lower than CV of empty-room noise between −10.5 and 0 cm on the *y*-axis plane (i.e., from the occipital cortex to precentral gyri; Fig. 3*A*). In this significant spatial range, APF deviated by as much as −2.2 to +1.7 Hz from the median in the physiological signal (Fig. 3*B*). To evaluate the integrity of anterior APF estimates, we performed a control analysis in which we excluded epochs if the identified APF did not exceed the threshold derived from empty-room recordings. We found a substantial reduction in both the number of retained epochs and the CV values of the anteriorly located parcels (Extended Data Fig. 3-2). Such that only ∼7% of epochs were retained in the frontal cortices, whereas ∼34% of epochs were retained in the occipital cortices. Notably, the linear increase in CV values along the posterior-to-anterior axis was still evident (*R*^2^ = 0.359, *p* < 0.001; Extended Data Fig. 3-2).

10.1523/ENEURO.0118-25.2025.f3-1 Figure 3-1 **Amplitude of simulated peaks** Log power of APF of physiological signal (in teal) and simulated signal (in blue) is plotted along the y-coordinate plane. Download Figure 3-1, TIF file.

10.1523/ENEURO.0118-25.2025.f3-2 Figure3-2 **Control analysis: SNR-based thresholding of APFs** The CV of physiological signal (in teal) and SNR-filtered signal (in green) is plotted along the Y-coordinate plane. The shaded region indicates the standard deviation across datasets. We found that the Y-coordinate position of the parcel significantly predicted the CV of the SNR-filtered APFs (R^2^ = 0.359, p < 0.001). Download Figure3-2, TIF file.

**Figure 4.**
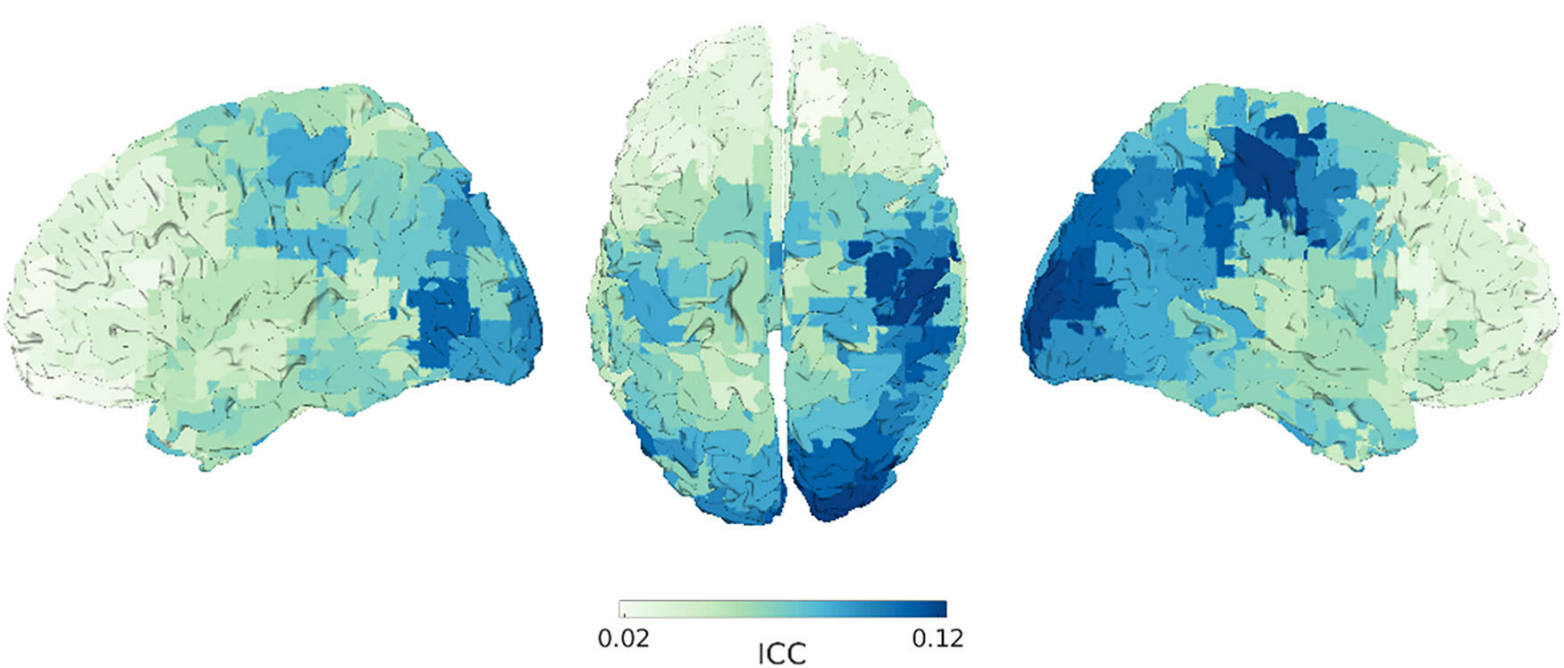
Estimates of intraclass correlation (ICC). ICC values of each parcel are projected on an MNI template brain. Lower estimates of ICC indicate poor reliability, whereas higher estimates are indicative of better reliability. Overall, APF shows poor reliability across the cortex reflecting substantial shifts in APF throughout. Distribution of APFs in the left lateral occipital cortex across participants (Extended Data Fig. 4-1) shows that low ICC values may reflect limited consistency within individuals and a lack of systematic differences between individuals. ICC estimates for 2 s (Extended Data Fig. 4-2) and 5 s (Extended Data Fig. 4-3) epochs reveal highest reliability in occipital regions and lowest in frontal regions.

ICC estimates at each of the parcels showed the highest reliability in right precentral (ICC = 0.121, 95% CI: 0.075–0.216) and postcentral (ICC = 0.122, 95% CI: 0.075–0.289) gyri and the left lateral occipital cortex (ICC = 0.123, 95% CI: 0.076–0.219). The right superior frontal gyrus (ICC = 0.018, 95% CI: 0.009–0.040) and left middle frontal gyrus (ICC = 0.019, 95% CI: 0.010–0.042; Fig. 4) had the lowest reliability. Overall, ICC values across the cortex were indicative of poor reliability of APF over epochs. The distribution of APFs in the left lateral occipital cortex of individual participants (Extended Data Fig. 4-1) indicate that low ICC values may reflect both limited consistency within individuals and a lack of systematic differences between individuals. Despite averaging power over longer segments of time (2 and 5 s), even regions with the highest ICC estimates (right lateral occipital cortex; ICC = 0.358, 95% CI: 0.244–0.531) fell short of having adequate reliability (i.e., ICC > 0.5; Extended Data Figs. 4-2, 4-3). Consistently across all epoch lengths, ICC estimates were highest in occipital regions and lowest in frontal regions.

10.1523/ENEURO.0118-25.2025.f4-1 Figure 4-1 **Distribution of APFs in the left lateral occipital cortex** *Histogram of individual alpha peak frequencies (APFs) across all participants (n = 24). The x-axis shows APF values in Hz, and the* y-axis indicates the number of epochs in which each frequency was identified as the APF in the left lateral occipital cortex. Download Figure 4-1, TIF file.

10.1523/ENEURO.0118-25.2025.f4-2 Figure 4-2 **Estimates of Intra-Class Correlation (ICC) from 2 s epochs** Parcel-wise ICC values are projected onto an MNI template brain. The lowest reliability was observed in the right middle frontal gyrus (ICC = 0.024, 95% CI: 0.011–0.056), while the highest was found in the left ventromedial occipital cortex (ICC = 0.207, 95% CI: 0.133–0.345). The colour bar is scaled to the maximum ICC value observed across parcels. Download Figure 4-2, TIF file.

10.1523/ENEURO.0118-25.2025.f4-3 Figure 4-3 **Estimates of Intra-Class Correlation (ICC) from 5 s epochs** Parcel-wise ICC values are projected onto an MNI template brain. The lowest reliability was observed in the right middle frontal gyrus (ICC = 0.056, 95% CI: 0.019–0.134), while the highest occurred in the right lateral occipital cortex (ICC = 0.358, 95% CI: 0.244–0.531). The colour bar is scaled to the maximum ICC value observed across parcels. Download Figure 4-3, TIF file.

## Discussion

In recent years, APF has gained considerable attention as a potential marker of human cognition. On the one hand, APF is highly heritable and resistant to change over test–retest intervals, but on the other, it is extremely volatile and susceptible to changes in task demands and shifts in intrinsic states (Mierau et al., 2017). In the present study, we showed that such peak frequency transitions of APF occur at small timescales (i.e., within 1 s) and the degree of variation in APF systematically increased from the caudal to rostral regions. We established that the observed fluctuations and the spatial gradient were not just an anomaly related to underlying noise. Further, we found that APFs show poor reliability across time over the entire cortex.

First and foremost, we confirmed that APF decreases along the posterior-to-anterior axis in a systematic fashion (in line with Mahjoory et al., 2020). The spatial gradient is governed by differences in the geometry of the cortex, in terms of cytoarchitecture and connectivity (Huntenburg et al., 2018), which in turn constrain the emergent dynamics (Demirtaş et al., 2019; Shafiei et al., 2023). The temporal dynamics of APF, however, has been largely overlooked. Therefore, we characterized the transient variations in APF to assess whether it more closely resembles a state than a trait marker. Our results illustrate that, not only APF, but also the degree of variation in APFs follows the posterior-to-anterior gradient, such that fluctuations in APFs exacerbates along this direction. This is in line with the findings of Salinsky et al. (1991) who provided evidence of variability on a similar timescale, but to a much smaller degree. Recent findings reveal that the APF shows temporal variations on a subsecond scale, evidently in the anterior regions (Smith et al., 2024), congruent with our findings. The temporal features of regional signals are likely governed by the anatomical and functional connectivity patterns with other cortical and subcortical regions. The caudal end of the gradient houses the lower sensorimotor functions, whereas the rostral end oversees higher cognitive functions. In effect, along the posterior-to-anterior direction, there is an increase in strength of synaptic excitation (Wang, 2020) and density of neurotransmitter receptors (Luppi et al., 2022), lengthening of timescales of information processing (Honey et al., 2012; Gao et al., 2020), and weakening of the coupling between structure and function (Larivière et al., 2020). By virtue of its neurobiological substrate, the rostral regions exhibit prolonged and flexible patterns of activity which complements its function, requiring the synergistic integration of diverse inputs (Suzuki et al., 2025). The results of our study are congruent in that respect. APF, reflecting intrinsic neural timescales, is higher in the caudal pole, whereas variations in APF, indexing input stochastics (Lefebvre et al., 2015) and neural malleability (Garrett et al., 2013), is higher in the rostral pole.

We found the signal-to-noise ratio (SNR) systematically decreased along the posterior-to-anterior axis, in relation to variations in APF. The alpha rhythm dominates the parieto-occipital region, whereas beta (13–30 Hz) and theta (4–8 Hz) oscillations are prominent in the sensorimotor and frontal regions, respectively (Groppe et al., 2013; Keitel and Gross, 2016). Due to the variation in dominant peak frequency across the cortex, SNR varies on par. Given that low power precludes the true estimation of APF, the variation in APFs along the posterior-to-anterior axis may simply reflect an increment in noise/or a reduction in alpha power. However, if that were to be the case, we would not expect to see a gradient in the parieto-occipital region.

We substantiated the variation in APFs along the gradient in comparison with noise, which was recorded from an empty magnetically shielded room. These findings reaffirm that there is a notable amount of variation in APFs from the occipital to the pericentral regions, following the gradient, which is not seen in empty-room noise. On adding a 10 Hz rhythm to empty-room noise, we found that the variation in empty-room “APFs” was much lesser than APFs observed in the physiological data, despite matching the amplitude of the simulated alpha and beta rhythms. This leads us to believe that the variation in APFs did not arise from random fluctuations in measurement noise. In the frontal regions, however, the amount of variation was indistinguishable from non-neuronal noise, with the power of identified peaks being comparable to the spurious peaks observed in the empty-room data. Therefore, we cannot ascertain whether the degree of variation in the frontal regions is a manifestation of the intrinsic neural dynamics. Due to the highly variable dynamics of the frontal regions, it is advisable to exercise caution in conventional analyses, as estimates of alpha phase and coupling indices may also be distorted by poor SNR (Aru et al., 2015; Donoghue et al., 2022).

Further, we found that estimates of reliability, as indexed by intraclass correlations (ICC), were poor across the entire cortex in contrast to previous studies. A low ICC could be indicative of low degree of agreement between measurements (i.e., substantial variability in APFs over epochs) or a lack of difference in APFs between individuals (Koo and Li, 2016). Kondacs and Szabó (1999) showed that APF has excellent reliability on comparing odd and even epochs of the same EEG session. Another study found good reliability across two measurements, repeated over the span of 10 months (Gasser et al., 1985). However, this study included a sample of children, and as APFs and variability in neural signals change over the course of development (Mišić et al., 2010), it does not offer a direct comparison. In addition, these studies used scalp recordings, which are inadequate to identify the sources of alpha activity due to spatial mixing of signals (Schaworonkow and Nikulin, 2022). Notably, in our dataset, averaging power spectra over longer epochs drastically improved ICC estimates, but the observed values did not meet the threshold for adequate reliability (Koo and Li, 2016). Therefore, our findings challenge the reliability of APFs and suggest that it may not be a stable neurophysiological marker that qualifies as a trait.

Our results challenge the common assumption that APF represents a stable, trait-like characteristic—a premise that underlies many fixed-frequency stimulation protocols. Stimulation paradigms targeting anterior alpha oscillations may be particularly vulnerable to temporal variability, potentially failing to entrain neural oscillations. Furthermore, the dynamic nature of alpha activity raises critical concerns for closed-loop stimulation approaches. If the temporal resolution of such systems fails to capture the rapid shifts in endogenous oscillatory parameters, stimulation may be mismatched with the targeted neural dynamics, thereby compromising efficacy. In our study, participants were instructed to be seated such that the back of their head is leaning against the fixed helmet of the MEG; therefore, the occipital cortex lies closest to the SQUID sensor array, whereas the proximity of the frontal regions to the sensor array varies between individuals. As a result, the magnitude of the signal, in relation to background noise originating from non-neuronal sources, is much higher in the posterior regions. However, the low reliability of APFs cannot simply be attributed to measurement inaccuracies because despite good SNR, and a relatively low degree of variability in the occipital region, the ICC estimates were not adequate (Boutros et al., 1991). Moreover, since we only analyzed datasets from a single measurement session, we cannot comment on the trait-likeliness of APF. Future studies would benefit from characterizing the spatiotemporal variations in APF over multiple sessions.

In general, the spectral parameters of alpha oscillations appear to be nonstationary over time. Specifically, we showed that the degree of variation in APF increases along the posterior-to-anterior direction. From the occipital to the pericentral regions, the variation in APFs was evident in a substantial number of epochs, but still to a lesser extent than what is observed in empty-room measurements, suggesting that the observed gradient is not a feature of measurement noise. In the frontal regions, however, the degree of variation was indistinguishable from noise, which highlights the necessity to consider temporal variability and overall signal-to-noise ratio in the study of alpha oscillations. Further, the poor ICC estimates obtained in our data suggest that, depending on the timescale of the investigation, APF could transition rapidly and to a similar extent across individuals, behaving more like a state than a trait.

## Synthesis

Reviewing Editor: James Howard, Brandeis University Decisions are customarily a result of the Reviewing Editor and the peer reviewers coming together and discussing their recommendations until a consensus is reached. When revisions are invited, a fact-based synthesis statement explaining their decision and outlining what is needed to prepare a revision will be listed below. The following reviewer(s) agreed to reveal their identity: NONE.

The authors have addressed some of the reviewers' prior concerns. However, the reviewers have ultimately determined that the reported findings do not make sufficiently substantive contributions to the literature. The result replicated Mahjoory et al. 2020 on the spatial distribution of APF. The primary analysis yielded a posterior-to-anterior gradient of APF fluctuation magnitude implied by Smith et al. 2024. However, the APF determination in anterior regions was likely poor (as discussed by the authors in the manuscript), potentially due to intrinsic limitations of MEG and/or the specific way their participants placed their heads within the MEG helmet. The reliability of APF was poor for 1s intervals but substantially improved for 5s intervals (nearing an acceptable level of reliability in some regions), implying that APF would likely be reliable beyond a reasonably short interval (e.g., 10s). It is unclear why the authors claim that APF is unreliable rather than determining the interval beyond which it becomes reliable. In summary, the reviewers find that the revised manuscript is mainly descriptive, and provides insufficient new insights, inconclusive inferences, and potential issues with participants' head placement in the MEG apparatus. Specific comments to the authors' rebuttal are as follows:

1. Smith et al. (2024) showed that spatiotemporal fluctuations of APF primarily reflected APF fluctuations in anterior regions, implying that APF fluctuations are greater in anterior than posterior regions. Moreover, Smith et al. 2024 also analyzed APF at short timescales. These results substantially overlap with the primary finding of the current study, limiting the impact of the finding.

2. The statements in the revised manuscript claiming that "the prevailing assumption is that APF remains stationary within and across experimental sessions," and "The temporal dynamics of APF, however, have been largely overlooked," are overly subjective and make assumptions about what scientists currently believe.

3. The reviewers find it difficult to grasp what can be inferred from analyzing the correlation between the CV and SNR over the posterior-anterior axis. For example, if there was a single dominant alpha peak (yielding a high SNR based on the authors' definition of SNR), APF fluctuations (CV) would reflect the peak-frequency fluctuations of the dominant peak. If there were a few prominent alpha peaks (thus, yielding low SNR), APF fluctuations (CV) could either reflect (1) the peak-frequency fluctuations of the highest peak (if it was stably higher than the other prominent peaks), (2) APF switching between the prominent peaks due to changes in the relative height of those peaks, or (3) APF switching between spurious peaks mostly generated by noise due to low alpha oscillation power relative to noise. The cases (2) and (3) yielding lower SNRs would generate higher CVs (as APF would switch between different peaks). While the authors infer (3) (179-180: "This suggests that low alpha power in the anterior regions may preclude the estimation of APF, resulting in seemingly high CV"), (2) would also be a possibility.

4. The authors state in a revised section (186-190): "To evaluate the integrity of anterior APF estimates, we performed a control analysis in which we excluded epochs with insufficient SNR, in relation to the noise distribution from empty-room data. We found a substantial reduction in both the number of retained epochs and the CV values of the anteriorly located parcels (Fig. 3-2)."

It appears that "SNR" here refers to the APF amplitudes of the physiological data relative to those of the empty-room data, rather than the amplitude of APF divided by the amplitudes of other peaks (the definition authors provide in the manuscript). Second, and more importantly, is the magnitude of APF fluctuation (CV) still significantly correlated with the posterior-anterior position in the flattened (above-noise-data-only) version shown in Fig. 3-2?

5. The authors provide the following statements in their revision: "Notably, most of the identified peaks in anterior regions failed to meet the minimum peak height criterion." "These findings raise the possibility that SNR in anterior regions is insufficient for reliable peak estimation, potentially leading to the detection of spurious peaks even in the real data." "In the frontal regions, however, the amount of variation was indistinguishable from non-neuronal noise, with the power of identified peaks being comparable to the spurious peaks observed in the empty-room data."

The result appears to imply that APFs obtained from anterior regions in MEG are unreliable. Are the authors suggesting that MEG is generally incapable of reliably detecting APFs in anterior regions? Are they thus implying that we cannot trust prior MEG studies that examined APFs in anterior regions? This is a very substantial and far-reaching claim. Would it be possible that the MEG apparatus used by the authors is noisier than others? In fact, the authors state in the discussion section that "In our study, participants were instructed to be seated such that the back of their head is leaning against the fixed helmet of the MEG; therefore, the occipital cortex lies closest to the SQUID sensor array, whereas the proximity of the frontal regions to the sensor array varies between individuals." Moreover, Jawata et al. (2023) systematically evaluated the reliability of MEG-detected oscillatory activity against intracranial EEG, and did NOT find that MEG was unreliable for detecting frontal oscillatory activity (though MEG tended to overestimate alpha oscillation amplitudes in frontal regions).

Jawata et al. (2023). Validating MEG source imaging of resting state oscillatory patterns with an intracranial EEG atlas. NeuroImage, 274.

6. The authors include the following statement in their revision: "Notably, in our dataset, averaging power spectra over longer epochs drastically improved ICC estimates, but the observed values did not meet the threshold for adequate reliability (Koo &Li, 2016)."

The authors now report that the ICC increased with interval length from 1, 2, to 5 sec, approaching 0.4 at 5 sec (at brain regions with maximal ICC). Given the sharp increase in the ICC up to 5 sec, it is inaccurate to claim that the ICC is unreliable. Since APF has been shown to be reliably associated with changes in psychological/physiological states (e.g., a review paper by Mierau et al., 2017 cited by the authors), it would be useful to evaluate longer intervals to determine the length sufficient to cross the "ICC>0.5" threshold. Given how close it is already with 5 sec, it is likely that the ICC would be reliable at 10 sec. Even if the interval had to be as long as, e.g., 30 sec, to cross the threshold, it would still be inappropriate to claim that ICC is unreliable for APF.

7. In the initial round of comments, the reviewers requested that the authors provide the actual variables (mathematical expressions) that go into the equation shown between lines 147 and 148 instead of the qualitative labels "variance of interest" and "unwanted variance."

The reviewers now note that the equation the authors present appears to be taken from McGraw &Wong (1996). This is fine, but the notations need to be appropriately modified. For instance, the references to rows (e.g., MSR) and columns (e.g., MSC) are meaningless here since they refer to the rows and columns in Table 2 of McGraw &Wong (1996).

8. The statement made in the abstract that systematic regional differences in alpha oscillations are "shaped by underlying signal-to-noise ratio" needs to be qualified to indicate that the caveat is specific to oscillatory neural activity measured with MEG. There is no evidence (of which the reviewers are aware) that suggests that EEG is also susceptible to a similar drawback. Moreover, the reviewers are not convinced of the authors' claim that MEG detection of anterior oscillatory activity is unreliable, given Jawata et al.'s (2023) systematic MEG-iEEG comparison, previous reports of anterior MEG alpha activity reliably correlated with behavior (e.g., working-memory related alpha-activity modulation in frontal-temporal regions), and the fact that the head placement in the authors' study was likely unfavorable for detecting anterior neural activity.

## Author Response

We thank the editorial team for allowing us to resubmit a revised version of our manuscript following our appeal. We will address the reviewer's comments in a point-by-point reply, citing reviewer comments in italic, our reply in normal font and revised text in the manuscript in bold.

Synthesis Statement for Author (Required): The authors have addressed some of the reviewers' prior concerns. However, the reviewers have ultimately determined that the reported findings do not make sufficiently substantive contributions to the literature. The result replicated Mahjoory et al. 2020 on the spatial distribution of APF. The primary analysis yielded a posterior-to-anterior gradient of APF fluctuation magnitude implied by Smith et al. 2024. However, the APF determination in anterior regions was likely poor (as discussed by the authors in the manuscript), potentially due to intrinsic limitations of MEG and/or the specific way their participants placed their heads within the MEG helmet. The reliability of APF was poor for 1s intervals but substantially improved for 5s intervals (nearing an acceptable level of reliability in some regions), implying that APF would likely be reliable beyond a reasonably short interval (e.g., 10s). It is unclear why the authors claim that APF is unreliable rather than determining the interval beyond which it becomes reliable. In summary, the reviewers find that the revised manuscript is mainly descriptive, and provides insufficient new insights, inconclusive inferences, and potential issues with participants' head placement in the MEG apparatus.

1. Smith et al. (2024) showed that spatiotemporal fluctuations of APF primarily reflected APF fluctuations in anterior regions, implying that APF fluctuations are greater in anterior than posterior regions. Moreover, Smith et al. 2024 also analyzed APF at short timescales. These results substantially overlap with the primary finding of the current study, limiting the impact of the finding.

In the first decision letter from April 23, it was stated that, "we find the work interesting and containing good science" and that "novelty is not a publication criterion for eNeuro, so it is not problematic that the major findings reported in this paper are strikingly similar to those reported in Smith et al., 2024". However, the paper has now been rejected despite our thorough revisions addressing all reviewer comments, with the reason being that "the work done thus far doesn't advance enough the field of neuroscience". We are in disagreement with the inconsistencies in publication criteria. The potential impact of the findings was not raised as a concern during the initial review round, yet it has become a determining factor at this stage.

2. The statements in the revised manuscript claiming that "the prevailing assumption is that APF remains stationary within and across experimental sessions," and "The temporal dynamics of APF, however, have been largely overlooked," are overly subjective and make assumptions about what scientists currently believe.

Following reviewers' suggestion from the first round, we rephrased the above-mentioned sentences to account for the findings of Smith et al., (2024). The use of phrases such as, "prevailing assumption" and "largely overlooked" indicate that most studies to date have not examined the short-lived dynamics of APF, and analyses are based on the assumption that APF is a stable trait-like marker.

To further clarify our position, we have rephrased the following sentence. (Significance statement) "However, the stability of APF within or across experimental sessions is seldom examined." 3. The reviewers find it difficult to grasp what can be inferred from analyzing the correlation between the CV and SNR over the posterior-anterior axis. For example, if there was a single dominant alpha peak (yielding a high SNR based on the authors' definition of SNR), APF fluctuations (CV) would reflect the peak-frequency fluctuations of the dominant peak. If there were a few prominent alpha peaks (thus, yielding low SNR), APF fluctuations (CV) could either reflect (1) the peak-frequency fluctuations of the highest peak (if it was stably higher than the other prominent peaks), (2) APF switching between the prominent peaks due to changes in the relative height of those peaks, or (3) APF switching between spurious peaks mostly generated by noise due to low alpha oscillation power relative to noise. The cases (2) and (3) yielding lower SNRs would generate higher CVs (as APF would switch between different peaks). While the authors infer (3) (179-180: "This suggests that low alpha power in the anterior regions may preclude the estimation of APF, resulting in seemingly high CV"), (2) would also be a possibility.

As noted in our previous revision, we have already addressed this concern through a control analysis. We acknowledge that our definition of SNR allows for multiple interpretations-changes in SNR could indeed reflect either the presence of multiple peaks or a reduction in peak power along the posterior-to-anterior axis. Consequently, the variation in CV values is likely multi-faceted and could arise from scenarios (1), (2), or (3). Importantly, this analysis demonstrates that CV varies in close correspondence with SNR. Moreover, the control analysis (Fig. 3-2) clarifies that there is a reduction in CV, on excluding epochs that do not meet the amplitude threshold, which is indicative of scenario (3). To address both possibilities, we rephrased the statement as follows. (II 182-184, p. 8) "Alternatively, it could also be driven by the presence of multiple alpha-band oscillations of comparable magnitude occurring simultaneously in anterior regions, with APF switching between peaks as their relative prominence fluctuates." 4. The authors state in a revised section (186-190): "To evaluate the integrity of anterior APF estimates, we performed a control analysis in which we excluded epochs with insufficient SNR, in relation to the noise distribution from empty-room data. We found a substantial reduction in both the number of retained epochs and the CV values of the anteriorly located parcels (Fig. 3-2)." It appears that "SNR" here refers to the APF amplitudes of the physiological data relative to those of the empty-room data, rather than the amplitude of APF divided by the amplitudes of other peaks (the definition authors provide in the manuscript). Second, and more importantly, is the magnitude of APF fluctuation (CV) still significantly correlated with the posterior-anterior position in the flattened (above-noise-data-only) version shown in Fig. 3-2? Indeed, we agree that it is critical to test whether the y-coordinate position still predicts CV after excluding the APFs that do not meet the noise threshold. We include the following statements to clarify the control analysis and report the new findings: (II 149-151, p. 7) "To test whether the regional differences in CV persisted after excluding APFs with low amplitudes, we performed a linear regression between the recalculated CV values and their corresponding Y-coordinate position." (II 191-192, p. 8) "... we performed a control analysis in which we excluded epochs, if the identified APF did not exceed the threshold derived from empty-room recordings." (II 194-197, p. 9) "...only ~7% of epochs were retained in the frontal cortices, whereas ~34% of epochs were retained in the occipital cortices. Notably, the linear increase in CV values along the posterior-to-anterior axis was still evident (R2 = 0.359, p<0.001; Fig. 3-2)." 5. The authors provide the following statements in their revision: "Notably, most of the identified peaks in anterior regions failed to meet the minimum peak height criterion." "These findings raise the possibility that SNR in anterior regions is insufficient for reliable peak estimation, potentially leading to the detection of spurious peaks even in the real data." "In the frontal regions, however, the amount of variation was indistinguishable from non-neuronal noise, with the power of identified peaks being comparable to the spurious peaks observed in the empty-room data." The result appears to imply that APFs obtained from anterior regions in MEG are unreliable. Are the authors suggesting that MEG is generally incapable of reliably detecting APFs in anterior regions? Are they thus implying that we cannot trust prior MEG studies that examined APFs in anterior regions? This is a very substantial and far-reaching claim. Would it be possible that the MEG apparatus used by the authors is noisier than others? In fact, the authors state in the discussion section that "In our study, participants were instructed to be seated such that the back of their head is leaning against the fixed helmet of the MEG; therefore, the occipital cortex lies closest to the SQUID sensor array, whereas the proximity of the frontal regions to the sensor array varies between individuals." Moreover, Jawata et al. (2023) systematically evaluated the reliability of MEG-detected oscillatory activity against intracranial EEG, and did NOT find that MEG was unreliable for detecting frontal oscillatory activity (though MEG tended to overestimate alpha oscillation amplitudes in frontal regions).

Jawata et al. (2023). Validating MEG source imaging of resting state oscillatory patterns with an intracranial EEG atlas. NeuroImage, 274.

Based on the findings of our study, we cautiously raised concerns about spurious peaks in the anterior regions that could distort the estimation of peak frequencies. Accordingly, we included the following statements in the discussion: (II 262-266, p. 11) "...we cannot ascertain whether the degree of variation in the frontal regions is a manifestation of the intrinsic neural dynamics. Due to the highly variable dynamics of the frontal regions... estimates of alpha phase and coupling indices may also be distorted by poor SNR (Aru et al., 2015; Donoghue et al., 2022)." (II 305-307, p. 12) "In the frontal regions, however, the degree of variation was indistinguishable from noise, which highlights the necessity to consider temporal variability and overall signal-to-noise ratio in the study of alpha oscillations." We would like to emphasize that these concerns are not specific to our MEG system. MEG sensors are embedded in a cryogenic dewar; hence their positions are fixed within a rigid "one-size-fits-all" helmet. As a result, the brain-to-sensor distance is inherently inhomogeneous, with the largest gaps typically found in frontal regions (Coquelet et al., 2020). These distances further increase in participants with smaller head sizes. Given that MEG signal strength decays approximately with the square of the distance from the source, signal quality is inevitably poorer in frontal regions. Importantly, this limitation is intrinsic to all cryogenic MEG systems and is only now being addressed with OPM-MEG.

Regarding the reference to Jawata et al. (2023), we believe that the reviewer misinterprets this study to support their position; on the contrary, their results largely support our findings. The reviewer states: "Jawata et al. (2023) systematically evaluated the reliability of MEG-detected oscillatory activity against intracranial EEG, and did NOT find that MEG was unreliable for detecting frontal oscillatory activity (though MEG tended to overestimate alpha oscillation amplitudes in frontal regions)." Framing it as 'tended to overestimate' is a clear misinterpretation, since Jawata et al. explicitly stated that MEG 'largely overestimated' alpha peaks. Moreover, Jawata et al clearly reported that the MEG detected peaks in frontal regions that were absent in iEEG. Thus, it appears that our results are completely in line with Jawata et al.

Relevant quotes from Jawata et al (2023) include: • "MEG largely overestimated oscillatory peaks in the alpha band, especially in the anterior and deep regions." • "We observe that MEG overestimated peaks in the alpha band for most ROIs, especially higher in frontal (lateral and medial) ROIs (>40%)" • "Our study also found that MEG-estimated spectra were dominated by oscillations in the alpha band, especially in anterior and deeper regions, unlike the actual in situ measurements from the atlas." • "Oscillatory peaks in the alpha band were largely overestimated by MEG, especially in frontal regions (Median ∼45-84%)." • "It is quite evident from intracranial EEG that alpha is not as prominent and widespread as seen from scalp recordings, especially not in the frontal regions." Coquelet, N., De Tiège, X., Destoky, F., Roshchupkina, L., Bourguignon, M., Goldman, S., ... &Wens, V. (2020). Comparing MEG and high-density EEG for intrinsic functional connectivity mapping. NeuroImage, 210, 116556.

6. The authors include the following statement in their revision: "Notably, in our dataset, averaging power spectra over longer epochs drastically improved ICC estimates, but the observed values did not meet the threshold for adequate reliability (Koo &Li, 2016)." The authors now report that the ICC increased with interval length from 1, 2, to 5 sec, approaching 0.4 at 5 sec (at brain regions with maximal ICC). Given the sharp increase in the ICC up to 5 sec, it is inaccurate to claim that the ICC is unreliable. Since APF has been shown to be reliably associated with changes in psychological/physiological states (e.g., a review paper by Mierau et al., 2017 cited by the authors), it would be useful to evaluate longer intervals to determine the length sufficient to cross the "ICC>0.5" threshold. Given how close it is already with 5 sec, it is likely that the ICC would be reliable at 10 sec. Even if the interval had to be as long as, e.g., 30 sec, to cross the threshold, it would still be inappropriate to claim that ICC is unreliable for APF.

We would like to reiterate that the objective of our study was "to characterise the non-stationarities in spectral parameters" (Abstract) and "to analyse the stability of the alpha peak frequency (APF) at short timescales" (Introduction). By definition, this necessitates analysing alpha peaks within brief temporal windows. The reviewers' suggestion to employ analysis windows of up to 30 seconds overlooks the central aim of our study. Naturally, extending the analysis window to, for instance, 20 minutes would yield more stable alpha peaks; however, such an approach would fundamentally deviate from our primary objective of characterising short-term fluctuations.

7. In the initial round of comments, the reviewers requested that the authors provide the actual variables (mathematical expressions) that go into the equation shown between lines 147 and 148 instead of the qualitative labels "variance of interest" and "unwanted variance." The reviewers now note that the equation the authors present appears to be taken from McGraw &Wong (1996). This is fine, but the notations need to be appropriately modified. For instance, the references to rows (e.g., MSR) and columns (e.g., MSC) are meaningless here since they refer to the rows and columns in Table 2 of McGraw &Wong (1996).

Thank you for your suggestion. We have modified the notations as follows. (II 159-164, p. 7) "...MSB is mean square between participants, representing the variance attributable to true differences between individuals... MSW is the mean square within participants..." 8. The statement made in the abstract that systematic regional differences in alpha oscillations are "shaped by underlying signal-to-noise ratio" needs to be qualified to indicate that the caveat is specific to oscillatory neural activity measured with MEG. There is no evidence (of which the reviewers are aware) that suggests that EEG is also susceptible to a similar drawback. Moreover, the reviewers are not convinced of the authors' claim that MEG detection of anterior oscillatory activity is unreliable, given Jawata et al.'s (2023) systematic MEG-iEEG comparison, previous reports of anterior MEG alpha activity reliably correlated with behaviour (e.g., working-memory related alpha-activity modulation in frontal-temporal regions), and the fact that the head placement in the authors' study was likely unfavourable for detecting anterior neural activity.

Thank you for your suggestion. We have modified the statement in the abstract as follows. (Abstract) "In general, our study elucidates the dynamic characteristics of alpha oscillations and reveals systematic regional differences which are, in part, shaped by underlying signal-to-noise ratio inherent to MEG recordings." We are confident that the concerns underlying the initial rejection arose from a misappraisal of our work. In this response, we have clarified the issues regarding head placement and shown that our conclusions are fully aligned with, rather than contradictory to Jawata et al. (2023).
