# Supplementary figures and images for: Spontaneous Fluctuations in Alpha Peak Frequency along the Posterior-to-Anterior Cortical Plane
Source: eNeuro. 2026 Jan 2;13(1):ENEURO.0118-25.2025. doi: 10.1523/ENEURO.0118-25.2025 (PMC12795306; doi:10.1523/ENEURO.0118-25.2025)

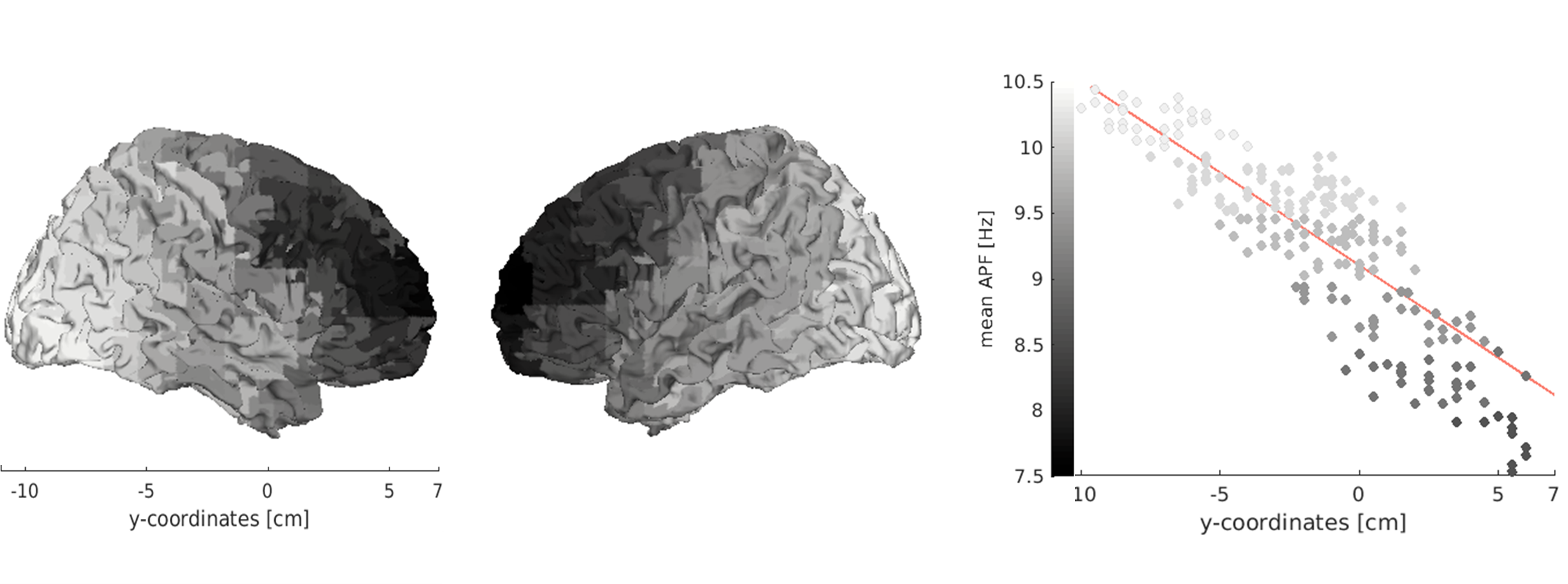

Supplement: Figure 1-1 — Distribution of time-averaged APF In the left panel, time-averaged APF for each parcel is projected on an MNI template brain. In the right panel, APF values were averaged along a spatially sliding window in the posterior-to-anterior direction. We found that the Y-coordinate position of the parcel significantly predicted APF (R2 = 0.646, p < 0.001). Download Figure 1-1, TIF file. [file eneuro-13-ENEURO.0118-25.2025-s001.tif]

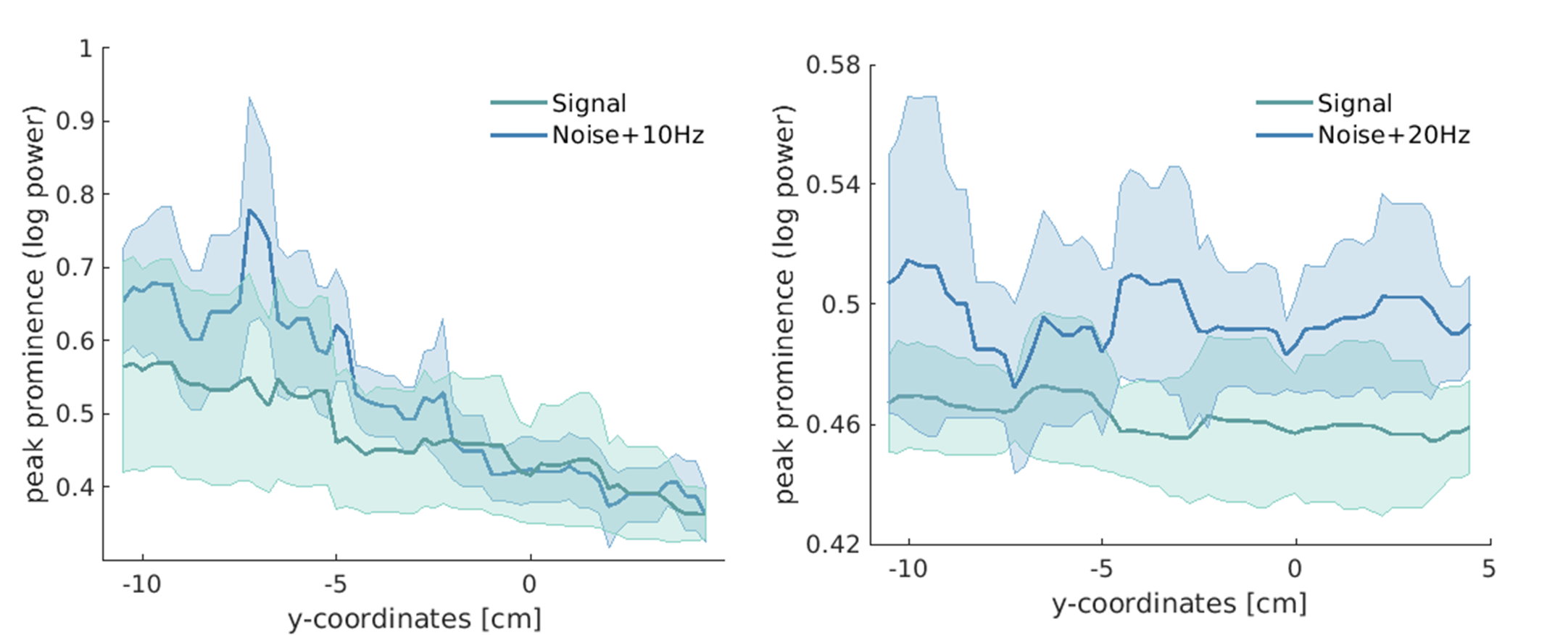

Supplement: Figure 3-1 — Amplitude of simulated peaks Log power of APF of physiological signal (in teal) and simulated signal (in blue) is plotted along the y-coordinate plane. Download Figure 3-1, TIF file. [file eneuro-13-ENEURO.0118-25.2025-s002.tif]

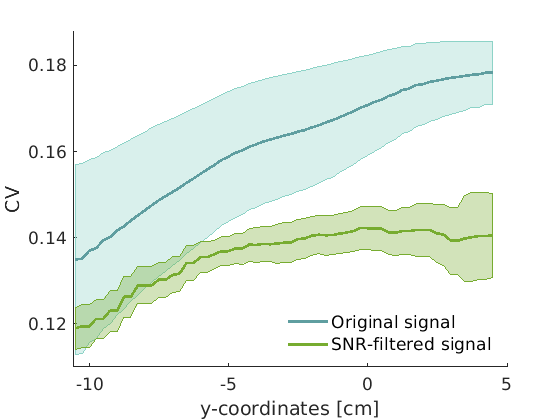

Supplement: Figure3-2 — Control analysis: SNR-based thresholding of APFs The CV of physiological signal (in teal) and SNR-filtered signal (in green) is plotted along the Y-coordinate plane. The shaded region indicates the standard deviation across datasets. We found that the Y-coordinate position of the parcel significantly predicted the CV of the SNR-filtered APFs (R2 = 0.359, p < 0.001). Download Figure3-2, TIF file. [file eneuro-13-ENEURO.0118-25.2025-s003.tif]

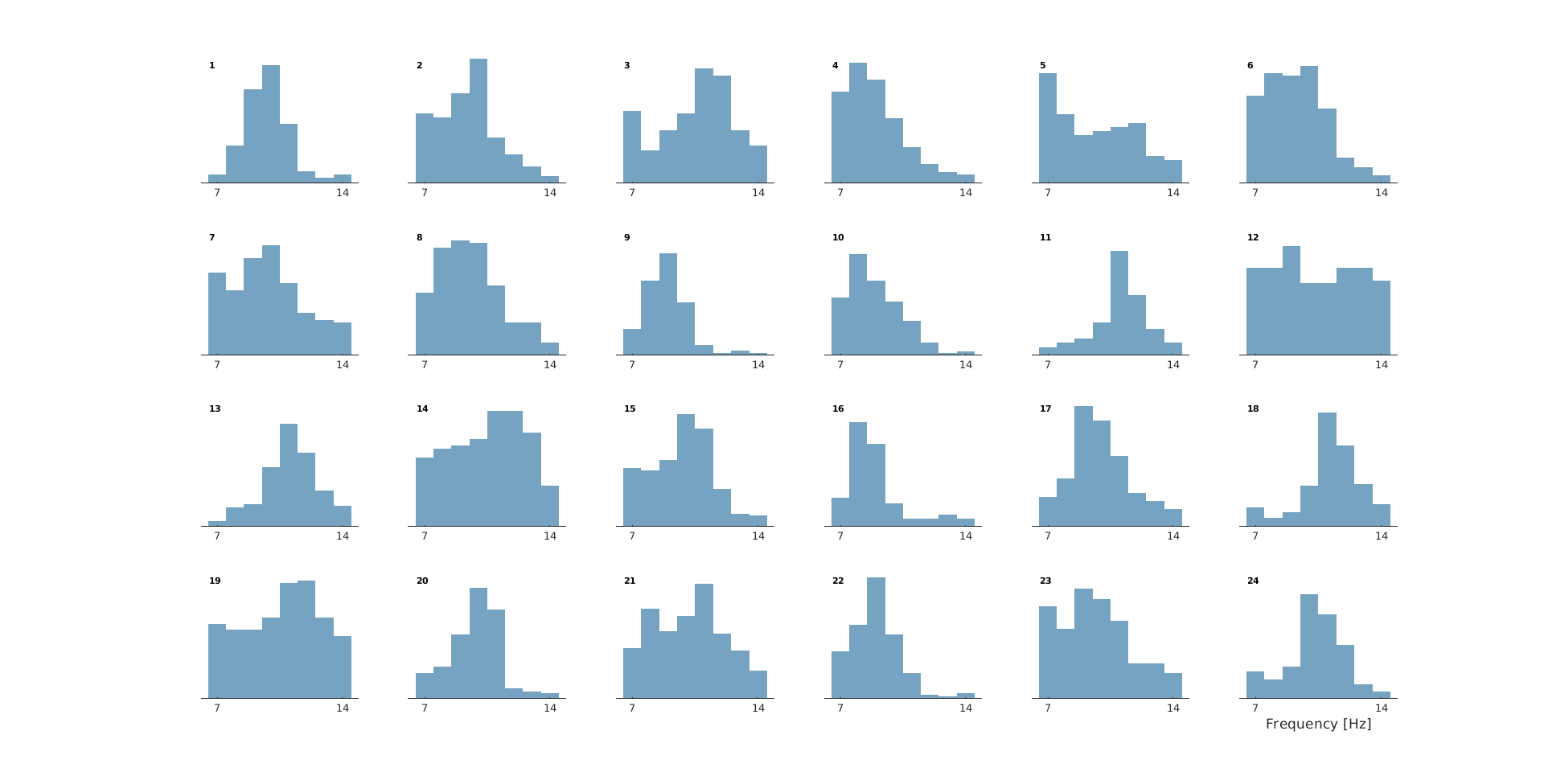

Supplement: Figure 4-1 — Distribution of APFs in the left lateral occipital cortex Histogram of individual alpha peak frequencies (APFs) across all participants (n = 24). The x-axis shows APF values in Hz, and the y-axis indicates the number of epochs in which each frequency was identified as the APF in the left lateral occipital cortex. Download Figure 4-1, TIF file. [file eneuro-13-ENEURO.0118-25.2025-s004.tif]

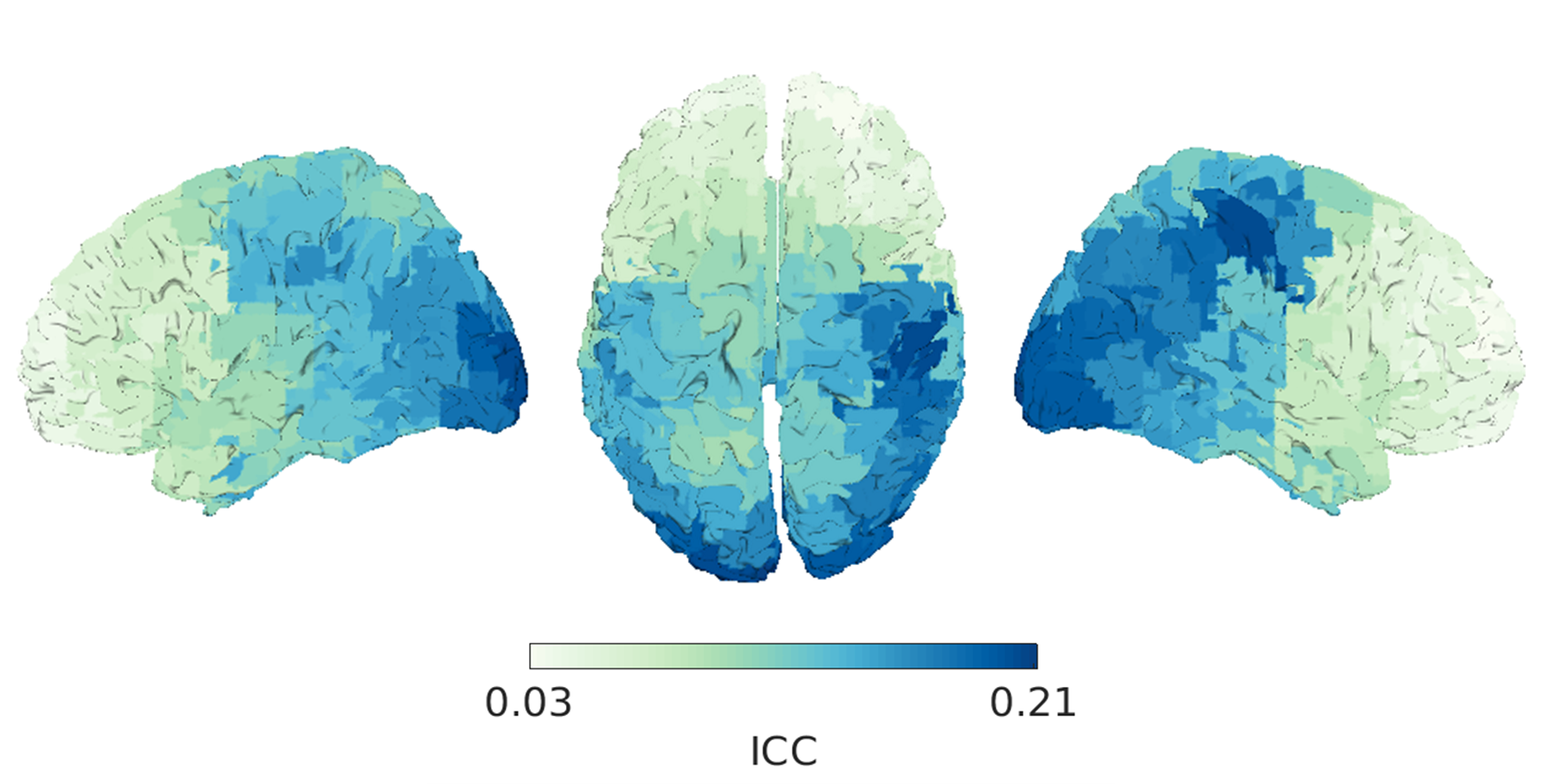

Supplement: Figure 4-2 — Estimates of Intra-Class Correlation (ICC) from 2 s epochs Parcel-wise ICC values are projected onto an MNI template brain. The lowest reliability was observed in the right middle frontal gyrus (ICC = 0.024, 95% CI: 0.011–0.056), while the highest was found in the left ventromedial occipital cortex (ICC = 0.207, 95% CI: 0.133–0.345). The colour bar is scaled to the maximum ICC value observed across parcels. Download Figure 4-2, TIF file. [file eneuro-13-ENEURO.0118-25.2025-s005.tif]

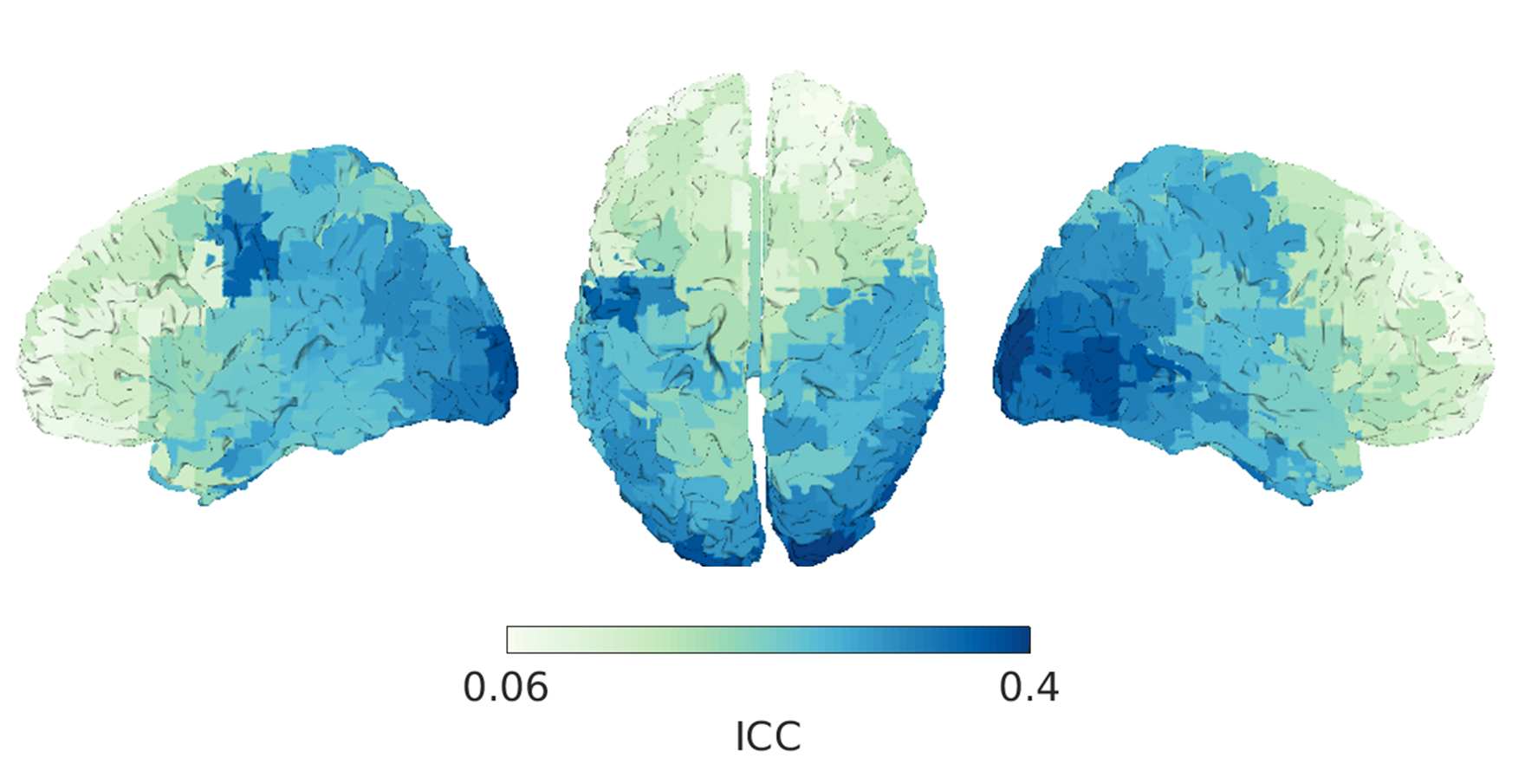

Supplement: Figure 4-3 — Estimates of Intra-Class Correlation (ICC) from 5 s epochs Parcel-wise ICC values are projected onto an MNI template brain. The lowest reliability was observed in the right middle frontal gyrus (ICC = 0.056, 95% CI: 0.019–0.134), while the highest occurred in the right lateral occipital cortex (ICC = 0.358, 95% CI: 0.244–0.531). The colour bar is scaled to the maximum ICC value observed across parcels. Download Figure 4-3, TIF file. [file eneuro-13-ENEURO.0118-25.2025-s006.tif]
